# Experimental and Numerical Analysis of Rotary Friction Welding for Al-Cu Joints: Effects of Friction Time on Plastic Deformation and Joint Integrity

**DOI:** 10.3390/ma18091932

**Published:** 2025-04-24

**Authors:** Nada Ratković, Dušan Arsić, Ružica R. Nikolić, Marko Delić, Živana Jovanović Pešić, Vesna Mandić, Jana Pastorková

**Affiliations:** 1 Faculty of Engineering, University of Kragujevac, 34000 Kragujevac, Serbia; nratkovic@kg.ac.rs (N.R.); marko.delic@kg.ac.rs (M.D.); zixi90@gmail.com (Ž.J.P.); mandic@kg.ac.rs (V.M.); 2 Research Centre, University of Žilina, 010 01 Žilina, Slovakia; jana.pastorkova@uniza.sk

**Keywords:** friction welding, aluminum, copper, friction time, friction pressure

## Abstract

The principles of the friction welding (FW) process of the two different non-ferrous metals, aluminum and copper, are presented in this paper. Considering that the bimetallic Al-Cu joints find applications in electrical engineering, as well as in other industrial fields, the basic characteristics and compatibility of these metals are discussed, along with the influence of various parameters on the properties of their friction welded joints. The experimental study involved rotation friction welding (RFW), which was used to weld aluminum and copper samples. The samples were monitored for shortening due to the applied deformation, as well as the size of the formed mushroom. Then, the central part of the welded joint was cut from the welded samples to determine the hardness and microstructure of the joint. At the end of the research, the possibility of applying software for the design of a numerical model for analysis of the possibility of joining aluminum and copper, with the same input parameters as those used in the experiment, was considered. The numerical simulation exhibited a high agreement with the experimental results.

## 1. Introduction

Welding of copper and aluminum using conventional welding techniques is highly challenging due to the high thermal diffusivity of these metals. Friction welding is often used as a solid-state welding technique to overcome this challenge. During friction welding, metals do not transform into the liquid phase; instead, they reach a visco-thermoplastic state in which, under pressure, permanent plastic deformation occurs, enabling the formation of a bond [1]. The most important advantage of friction welding is the fact that it achieves a perfect weld, that is to say an integral weld (weld the surface and not the circumference) because the parts are forged/welded into one set. Friction welding is carried out sustainably. The great advantage of this welding technique lies in the fact that some combinations of materials are easily welded, while they cannot be welded by traditional welding techniques [2]. The rotational friction welding (RFW) of various metals and corresponding analyses are given in [3,4,5,6,7]. Friction welding stands as a suitable welding method widely applied for joining Al and Cu in the electrical industry, thermo-technology, and various other applications across industrial practices [8,9] including application in the nuclear industry [10].

In addition to rotary friction welding (applied in this study for joining cylindrical elements), the development of advanced welding technologies and the focus of contemporary research also encompasses processes such as Friction Stir Welding (FSW) or Friction Stir Spot Welding (FSSW) welding (for joining plate or sheet metal components) [11,12,13].

The friction welding of aluminum and copper ensures a high-quality joint. The welding process is brief and cost-effective, as it requires no additional materials, gases, or chemicals, unlike other welding methods. Additionally, no extra energy is needed, and the minimum sample preparation before the welding is sufficient, as the process itself extrudes any residual oxides, oils, or other contaminants, ensuring that only the clean metals are in contact [8]. In addition, the friction welding process is environmentally friendly. Detailed microstructural research results on Al-Cu welded joints are presented in some previous research [14,15,16,17]. Paper [14] dealt with the microstructure analysis of steel, while papers [15,16,17] dealt with the structure of Al-Cu welded joints. In the paper [15], the authors dealt with welding dissimilar joints between AA7075 and AA2024 alloys, which showed increased flash formation on the AA7075 side due to its lower melting point relative to the AA2024 alloy. They identified various zones within the weld region, such as the dynamic recrystallized zone (DRZ), the thermo-mechanically affected zone (TMAZ)–which includes TMAZ-1 with elongated grains and TMAZ-2 with compressed or distorted grains—the heat-affected zone (HAZ), and the base metal (BM) zone. In the paper [16], the authors identified that on welded Al-Cu samples a continuous intermetallic compound layer of a thickness of 0.7–10 μm, parallel to the interface, is distinctly visible between the Al and Cu bulk. They found that the intermetallic layer increases with increasing welding time. In the paper [17], the authors concluded that Al samples contained large grains in the central region and smaller, elongated, and deformed grains in the surrounding areas. The Al microstructure was similar in the inner peripheral and near inner peripheral regions. The equivalent diameter of the largest Al grain was approximately 500 ± 200 μm. The Cu samples featured large grains, with the presence of twin boundaries and smaller equiaxed grains. Microhardness values of Al and Cu samples were determined to be 42.6 ± 3.9 and 93.1 ± 6.7 HV0.01, respectively.

Researchers in this field, during the parametric optimization of the friction welding process, consider friction time as the most influential factor, followed by friction pressure and compression, and rotational speed [7,18,19,20]. It has also been determined that these parameters directly impact the level of plastic deformation, mechanical characteristics of the welded joint and microstructural changes [21,22,23,24]. For instance, it was found that the friction time and friction pressure influence the tensile strength of the joint [5].

The friction welding process of copper and aluminum parts was, in this research, studied experimentally and by numerical simulation. The objective of this research was to analyze the possibilities for making a bimetallic joint that would have satisfactory strength, without the appearance of unwelded places or places with high hardness. The experimental section of this study involved the welding of samples, followed by preparing the samples for further examination of mechanical and microstructural characteristics. The influence of friction time on the shape and size of the obtained plastic deformation in these joints was analyzed, and the hardness values were measured in the joining zone. The biggest task in the research of joining different materials is to determine the optimal parameters when varying the process parameters and for given operating conditions. Compared to previous works, this work is more advanced due to numerical support. The practical significance is in predicting the length of the samples after the joining.

The numerical approach to the task is certainly an advanced step in this field of research. Numerical modelling is still not sufficiently represented in these works and each new and/or different approach is a step forward in achieving quality solutions. If a numerical model was successfully developed, that could later greatly help in solving certain problems in some future research, speed up the analysis process, and make it possible to bypass expensive and time-consuming experimental work, which would shorten the time of product development.

At the end of the presented research, the possibility of establishing a numerical model of the performed experiment was analyzed. The objective of this step was to optimize the numerical model so as to obtain results as similar as possible to the experimental ones, which were successfully performed in several earlier studies [25,26]. This is the main contribution—the novelty of the presented results. The minor deviation between the numerical simulation results and experimental results is proof that the proposed numerical model was properly designed and executed.

## 2. Literature Review

Since some of the common features of different groups of references used in this research are briefly explained in the introductory section, this literature review is focused only on some of the most important, characteristics of those groups.

Jin et al. 2023 [3] were studying the heat-pattern (HP) induced non-uniform radial microstructure and properties of the rotary friction welded Ti-6Al-4 V joint. They distinguished the two types of HP, the “glass-like” HP and the “scissor-like” HP. The former one results at low rotational speeds (500 to 1200 rpm), when the joint squeezes the interfacial plasticized metal towards and accumulates at the periphery, and the axial tensile stress is not sufficient to flip the metal into the extruded flash. The latter HP is formed, at higher rotational speeds (1500 to 1800 rpm), when the plasticized metal would flow to and would be extruded at the periphery. The non-uniform radial microstructures cause the non-uniform radial properties.

Kimura et al. 2014 [5] have analyzed the fracture of the friction-welded joint between pure nickel and aluminum with post-weld heat treatment (PWHT). The joint fracture temperature increased with increasing width of the intermetallic compounds (IMC) interlayer in the axial direction of the joint. The fracture of the joint occurred at the interface between the NiAl layer and Al base metal, due to a PWHT-induced decrease of the interface bonding strength. However, the joint fracture temperature drastically increased up to about 800 K when the width of the IMC interlayer exceeded 50 mm.

Liang et al. 2015 [6] studied the dissimilar friction welding of Al and Mg bars and evaluated the interfacial microstructure characteristics of the Al-Mg alloy using optical and scanning electron microscopy, and X-ray diffraction. Experimental results showed that intermetallic compounds (IMCs), were generated in the interfaces of the Al and Mg alloys, with the thickness of IMCs decreasing with the friction and forge pressure increase. Heavy thickness of IMC layers seriously deteriorated the mechanical properties of the joints, with hardness at the interfaces being higher than that of the base material due to the IMCs’ presence.

Shubhavardhan et al. 2012 [7] considered the development of the solid-state joints of dissimilar materials, AA6082 aluminum alloy and AISI 304 stainless steel, formed by the continuous drive friction welding process (CDFW). With increasing the friction pressure and friction time, the joint strength increased and then decreased after reaching the maximum. The presence of contaminants at the interface of the metals reduced the joint quality. The surface finish operations did not affect the welding properties significantly. The hardness of both materials in the vicinity of the weld interface was higher than that of the base metals.

Gavalec et al. 2023 [9] experimentally studied the effect of the contact surface geometry on the quality and mechanical properties of a rotary friction weld (RFW) of the titanium alloy Ti6Al4V, testing three types of geometry: flat on flat, flat on 37.5° and flat on 45°. Results have shown that the best was the flat geometry, since it resulted in the least saturation with interstitial elements from the atmosphere, while the other two geometries were found as not suitable for the RFW of the titanium alloy Ti6Al4V. The fracture in the RFW zone was brittle, while the pure ductile fracture occurred in the heat-affected zone (HAZ).

Dellepiane et al. 2025 [10] have presented the design process and functionality of a laboratory-scale friction welding setup, which was designed to use the load cell, which enabled monitoring of the welding parameters. A change in the hardness along the weld interface was recorded, as well as changes in the microstructure of the Ca104 alloy, which was ascribed to the grains experiencing dynamic recrystallization during the welding. The flexural strength of the welded joints was higher than that of the base material.

Singh et al. 2012 [11] have analyzed the influence of axial force on the mechanical and metallurgical properties of FSW joints of Al alloy AA6082-T65. The axial force was varied within the range of 3 to 8 kN on the surface of the base material. No macro structure defects were observed for the force values between 4 to 7 kN; the maximum weld tensile strength (266 MPa) was obtained for the axial force of 6 kN, which was 85% of the base metal value.

Veljić et al. 2024 [12] studied the FSW of plates made of the high-strength Al alloy 2024-T3 of 3 mm thickness, specifically the tool plunge stage, which precedes the formation of the T-joint. The authors pointed out that an increase in the tool rotation speed increases the temperature in the welding zone, creating conditions for eliminating the joining line, while the same effect was exhibited by an increase in the tool pin length.

Zhu et al. 2022 [13] investigated the continuous drive friction welding of 6061-T6 Al and copper and concluded that with an increase in rotation speed, the width of the welded zone gradually increases, with the generation of higher temperatures. The microhardness on the bonding surface was significantly greater than those of the base materials due to the presence of IMCs, while the softening zone was formed on both sides of the bonding surface, becoming more significant with an increase in the rotation speed.

Bauri et al. 2024 [15] explored the effects of various pre-weld and post-weld heat treatments (PWHTs) on the microstructural and mechanical properties of the rotary friction welded (RFW) joints of the two dissimilar aluminum alloys, AA7075 and AA2024. The joints exhibited increased flash formation on the AA7075 side due to its lower melting point. All welds failed in the heat-affected zone (HAZ) of the AA2024 alloy side, due to the development of coarse grains, pointing out that this area was the weakest link in the joint.

Wei et al. 2015 [16] analyzed the joints between Al and Cu bars obtained by continuous drive friction welding (CDFW). The interface layers’ temperatures were within range of 648 to 723 K, at different welding parameters. The interdiffusion between Al and Cu atoms was extraordinarily rapid, as the interdiffusion coefficients reached 7.8 × 10^−12^ m^2^/s.

Milašinovic et al. 2024 [17] aimed to enhance the efficiency and efficacy of the Al/Cu friction joint production process, by obtaining sound joints within a very short welding time. The authors investigated the accuracy and the quality of the continuous drive friction welding (CDFW) process, and the optimum combination of its parameters. The thermal imaging showed the actual total welding time was 15% longer than the nominal value. The Al/Cu joints produced at welding pressures below 88.9 MPa often displayed the presence of non-joined micro-regions at the Al/Cu interface. At the friction pressure set at 66.7 MPa, an increase in the forging pressure to 222.2 MPa eliminated the presence of non-joined micro-regions.

Tashkandi and Mohamed 2020 [18] have investigated the effect of friction time on the mechanical properties of AA6061 joints made by the CDFW. They found that AA6061 does not require a forging stage and the solid joints did not fracture within the welding zones. The thermal profiles of the welding times indicated that the peak temperature is reached within 3 to 4 s of the welding process regardless of the duration of the welding procedure. Any further increase of the friction time does not increase the temperature beyond the peak one, but helps annealing the material.

Mahajan et al. 2023 [19] investigated the changes in the microstructural and mechanical properties of various pre- and post-weld heat treatments (PWHTs) on rotary friction welded of AA7075 and AA5083 aluminum alloys. They focused on the evolution of the weld’s macro and microstructures, and changes in hardness and tensile properties. Due to the continuous dynamic recrystallization, significant grain refinement was observed at the weld interface, in the dynamic recrystallized zone–DRZ, while in thermo-mechanically affected zone TMAZ, the region next to DRZ, the lower strain and high temperatures resulted in the formation of deformed grains.

He et al. 2014 [20] have reviewed developments in the numerical analysis of friction stir welding processes, microstructures of friction stir welded joints and the properties of friction stir welded (FSW) structures. The authors discussed the main methods in the numerical analysis of friction stir welding and illustrated with brief case studies, as well as certain important numerical modeling issues, such as materials flow modeling, meshing procedure and failure criteria.

In Gite et al. 2019 [21], the objective was to summarize the prominent research work in the field of friction welding, with emphasis on the methodology, workpiece materials, tool design and material, workpiece sizes, as well as the typical process parameters. The authors discussed the FSW principle and process parameters, like the tool geometry, tool rotational speed, tool traverse speed, joint design, as well as welding parameters, with a short overview of numerical analysis of the FSW processes.

Sethi et al. 2021 [22] have presented a review on friction stir welding as a sustainable way of manufacturing. They analyzed publications on FSW of similar alloys (Al-Al), dissimilar alloys (Al-Mg), and effects of interlayer on similar alloys (Al/Cu/Al) and dissimilar alloys (Al/Zn/Mg). They also considered the effects of the process parameters on the joining of materials by the FSW.

Çam et al. 2022 [23] have shown that the main difficulty in friction stir welding/friction stir spot welding (FSW/FSSW) of dissimilar Al-alloys is the lack of homogeneous mixing of materials due to the insufficient heat input, which leads to the formation of inhomogeneous structure in the stir zone and low plasticity of the materials to be welded. For the successful FSW butt and lap joining of dissimilar Al-alloys one has to choose the optimum weld parameters, such as heat input, plate and tool positioning, and shoulder geometry, as well as the pin profile, while in the FSSW of these alloys, other parameters, such as tool plunge speed, tool plunge depth and dwell time are influential.

Kumar et al. (2025) [24] analyzed the possibility of producing the Cu/SiC surface composite via friction stir processing. That process does not allow temperature to reach the interfacial interaction between Cu and SiC. Authors employed the scanning electron microscopy, electron backscattered diffraction, and optical microscopy to characterize the composite microstructural features. Results indicated the presence of Cu and SiC phases in the stir zone (SZ) with uniform and homogeneous separation of reinforcements. The composite displayed higher hardness, tensile strength, and wear resistance in comparison to unprocessed copper.

As emphasized at the end of the previous section, the objective of this work was to establish the numerical model of the FRW process and validate it by comparison to the experimentally executed test weldings’ results. That, in some way, fills the gap in the previous (here only partially) presented research works.

## 3. Experimental Procedure

### 3.1. Materials

Copper and aluminum, due to their high electrical and thermal conductivity and corrosion resistance, have common applications in almost all the fields of modern technology, such as electrical conductors, elements of heating and cooling technology, architectural hardware, etc. Their common application often requires their joining, forming the bimetallic joints of certain structural elements. The joining of these two metals can be performed in two ways: with additional material (soft and hard soldering) and without additional material (friction welding, resistance welding, cold welding, ultrasonic welding, etc.).

**Figure 1:**
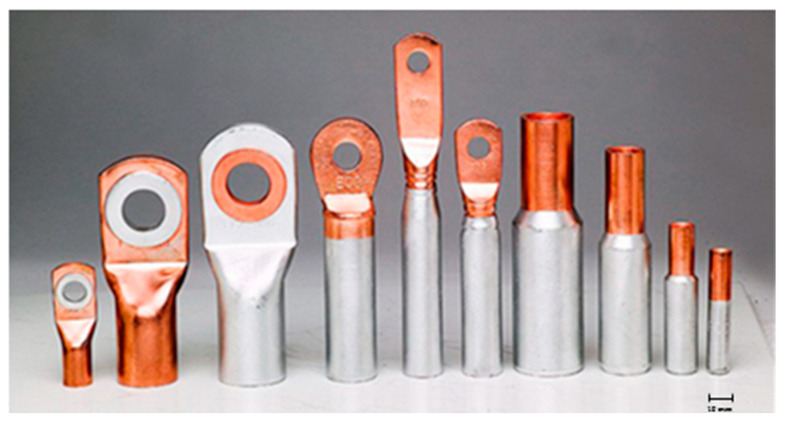
Copper-aluminum cable connectors [27].

In the electrical industry, a copper-aluminum terminal clamp, or a cable connector, is produced in certain cylindrical shapes. Examples are given in Figure 1 [27]. For the needs of the electrical industry, Al/Cu connectors, Al/Cu bimetallic cable lugs, Al/Cu bimetallic sleeves and Al/Cu bimetallic terminations are used; additionally, for connecting aluminum cables with multiple wires of different cross-sections to electrical devices or connections. What concerns the advantages of using this friction welding joining method, as compared to conventional methods, is a matter of the necessity of using this joining method. First of all, two different materials are joined with different thermo-physical and mechanical characteristics, so the use of conventional joining methods in industrial production is practically impossible.

These connectors are used where an aluminum cable needs to be terminated with a copper busbar or copper contact. If cable lugs made solely of copper or solely of aluminum were used, there would be a galvanic effect due to the contact of different metals, or metals with a significant difference in electrode potential. The electrode potential of Al is −1.662, and of Cu is +0.337. Whenever the two different metals come into contact, the so-called galvanic effect occurs due to their different electrode potentials, resulting in corrosion on the surface of the metal with a lower electrode potential, and in this case, that is aluminum. This is the electrochemical corrosion of different metals. To avoid this phenomenon, bimetallic lugs (connectors) are manufactured using friction welding. This ensures a technically sound and durable joint, fully suited for its intended purpose. Based on this, it can be said that aluminum and copper are compatible metals, considering certain properties and the quality of the obtained joint. However, compatibility does not apply to every aspect of these metals’ properties. For example, Al and Cu have mutual affinity at temperatures above 120 °C, forming several different common phases in their joint. These intermetallic phases negatively impact the mechanical properties of the joint and thus its reliability. A significant advantage of friction welding is that the process is relatively short, reducing the possibility of intermetallic phase formation.

The properties of the materials used in the experiment are given in Table 1.

### 3.2. The Mechanism of Al-Cu Joint Formation and Accompanying Formations During the Joining Process

Given the complexity of physical, mechanical, metallurgical, thermal, and chemical phenomena during the friction welding joining process of the two different metals, their joining mechanism is much more intricate than with homogeneous materials [9]. An alloy is formed between the surfaces of the two metals, and its properties can significantly influence the overall properties of the joint. The joining mechanism and the physical foundations of the joining process have been partially, but not fully explored. Therefore, there are still open questions that need to consider the influence of a series of relevant factors related to these processes and joining technology. In theory, there exist several hypotheses that define the joint formation process (diffusion, recrystallization, energy, dislocation, etc.), but none provides a complete solution, as the complexity of the answers is compounded by the fact that it involves welding of different metals.

The most acceptable theory is diffusion since the conditions for diffusion occur during the friction welding process, namely elevated temperatures and pressure.

The main characteristic of the joining mechanism in the friction welding process is that the metal contact surfaces need to approach a distance of the order of the crystal lattice parameter. Since the real metal surfaces are not perfectly smooth, contact at the beginning of welding occurs only at the peaks of the surfaces’ irregularities, and an increase in the contact area is achieved by the plastic deformation of the coupled peaks. When the peaks are completely compressed, the contact surface distance approaches the order of the crystal lattice parameter, at which point the atoms of one metal enter the crystal lattice of the other metal, and vice versa. Since the radii of aluminum and copper atoms are of similar size (for Al it is 0.143 nm and for Cu it is 0.128 nm), bonds are formed allowing for the formation of substitutional mechanism common crystal lattices. The joining is based on the formation of a metallic bond (solid solutions) between the base metals (aluminum and copper), all thanks to the diffusion phenomenon [1].

The interdiffusion between Al and Cu is accompanied by the formation of an intermetallic layer. The width of the diffusion layer is related to the diffusion time, and the diffusion time is a function of the friction time. For example, with a friction time of 8 s, the width of the diffusion layer can be up to 10 μm [14]. This intermetallic formation contains saturated solid solutions Al (Cu) and Cu (Al), which form on both sides of the contact surfaces due to mutual diffusion. The level of released heat, or the achieved temperatures in the contact zone, depends on the friction time. The measured temperatures in that zone range from 350 °C to 500 °C [4]. Diffusion occurs relatively quickly due to elevated temperatures and pressure, as the diffusion coefficient between Al and Cu atoms is 7.8 × 10^−12^ m^2^/s [6].

For definitive conclusions, researchers emphasize the necessity of a more detailed analysis of all the phenomena and formations accompanying the welding process. Given that it involves a two-component system, the microstructural processes during the heating of both metals become increasingly complex. During the heating process, resulting from the friction of the contact layers, the following equilibrium phases are formed: Al-Cu, Al_2_Cu, Al_3_Cu_4_, Al_2_Cu_3_, and Al_4_Cu_9_ [8]. Specific phases are formed in the temperature range from 370 °C to 450 °C. In the range of 350 °C to 500 °C, Al_2_Cu (θ-phase) and Al_4_Cu_9_ (δ-phase) are formed, particularly in the diffusion zones with a width exceeding 5 μm.

Intermetallic phases lead to a decrease in ductility, i.e., an increase in brittleness in the joint zone. Detailed research has shown that the intermetallic phase Al_2_Cu is formed the first. The Al_2_Cu phase segregates along the grain boundaries in the form of white, clustered deposits. In addition, it was established that this phase is formed on the aluminum side, while on the copper side, the Al_4_Cu_9_ phase is formed.

On the other side, the grain size reduction leads to an increase in the volume of the grain boundary, which can influence the material’s electrical conductivity. Furthermore, the authors in the paper [26] investigated the influence of adding graphene in the aluminum region which resulted in a slight increase in grain size. This increase helps to reduce the volume of the grain boundary, which in turn can limit the scattering of electrons. The presence of graphene abundantly in the aluminum region was beneficial for improving the material’s electrical conductivity. In addition to that, due to the thermal influence, the thermo-mechanical affected zone undergoes the dissolution of precipitates and has a grain size coarser than the base material due to the thermo-mechanic effect of the welding process. If the grain size is coarser, the total area of grain boundary per unit volume (that opposes electron mobility) is smaller, so, the electron mobility is easier to achieve and consequently, the electrical conductivity increases to about 17% relative to the base material [28,29].

### 3.3. Basic Features of the Applied Friction Welding Procedure

The friction stir welding is a solid-state joining process, as the weld is formed at a temperature lower than the melting temperature of the base metals. It belongs to the pressure welding processes. The joint is created by a combination of thermal and mechanical energy. Thermal energy is generated as a result of frictional heating between the welding elements, while mechanical energy is applied by the pressure force [4].

The experimental weldings were executed on the welding machine type MZT30-2 NC, “Potisje”, Ada, Serbia, with the following specifications: maximum axial friction force is 45 kN, axial compression force is 150 kN, speed up to 2500 rpm, diameters of rods that can be welded are from 10–30 mm, the maximum length of parts to be welded in the spindle 170 mm, in the support 450 mm.

**Figure 2:**
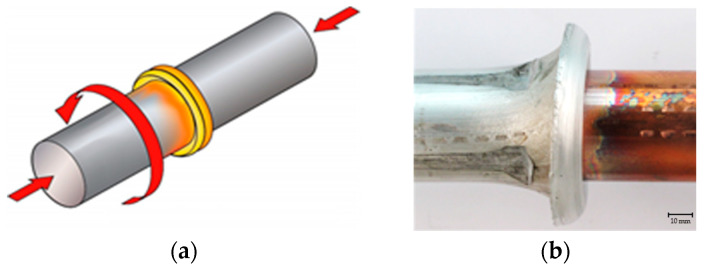
(**a**) Friction stir welding scheme; (**b**) Executed joint with extruded material (Al)—“mushroom”.

The procedure is carried out by rotating one part (held in the jaws of the welding machine) while the other part is firmly fixed on a slider. The part mounted on the slider cannot rotate but can move linearly to establish contact and generate friction pressure. When the end faces of the parts to be joined come into contact, the friction begins. As a result of the friction, a significant amount of heat is generated, which heats both parts and brings them to a plastic state [13]. The rotational movement is then abruptly stopped, ceasing further heat generation. An axial compressive force is then applied to the heated and softened parts, ensuring joining and welding, Figure 2a. Friction pressure induces maximum plastic deformation in both the axial and radial directions. In the axial direction, the parts are shortened, while in the radial direction, the material is extruded along the perimeter, forming a material ridge or the so-called “mushroom” shape, Figure 2b.

Since aluminum has a lower strength than copper, the aluminum part achieves a greater degree of plastic deformation in the radial direction as the material is extruded along the perimeter. Given that the material is heated by an internal heat source, by maintaining the temperature of the contact layers below the melting point of the more fusible metal (aluminum), and the released heat being strictly localized, a narrow heat-affected zone (HAZ) is formed.

A significant advantage of friction stir welding, compared to conventional welding methods is that the process takes a relatively short time with low energy consumption. However, metal joining is achieved only within a narrow range of welding parameters.

The nature of the aluminum and copper structure provides stronger heat release effects during the friction process, i.e., the energy required for the joint formation. It has been proven that during the friction of dissimilar metals with a face-centered cubic (FCC) crystal lattice structure, the friction coefficient is higher compared to the friction of dissimilar metals with a body-centered cubic (BCC) crystal lattice structure. This fact directly indicates that the friction between Al and Cu allows for the rapid generation of the energy required and is sufficient for welding, as these metals are excellent heat conductors.

### 3.4. Experimental Investigations of Plastic Deformation Flow and Its Dependence on Friction Time

During the welding process, rapid and intense deformation occurs. Plastic deformation is complex and initially occurs on the micro-scale irregularities of aluminum (as aluminum is more ductile than copper), before spreading to deeper layers. As a result of thermomechanical processes in the contact layers, plastic deformation intensifies rapidly. The intensity of plastic deformation depends on the technological parameters of the process, such as friction time, pressure time, or the total welding time, as well as the welding speed (number of rotations), and the corresponding friction and compression pressures.

Plastic deformation is on a micro-scale manifested as the formation and rupture of micro-joints, as well as the deposition of copper layers on the aluminum contact surface, leading to the formation of a mixing zone, causing the friction plane to deviate from the joint plane. Additionally, in the final dimensions, there is a change in the dimensions of the welded parts (length and diameter) at the joint [14].

**Figure 3:**
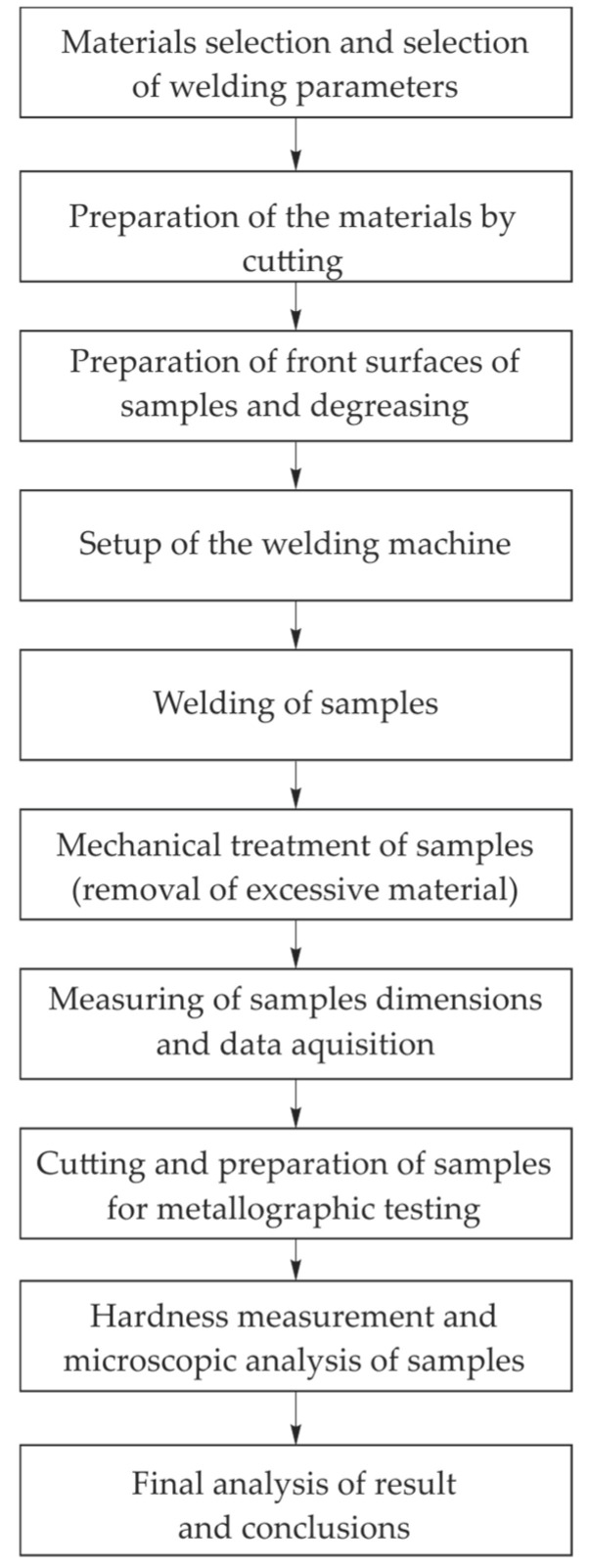
The flowchart of the experimental procedure.

The experimental plan included the preparation of samples to the appropriate dimensions, their welding, measurement of newly formed dimensions, preparation of samples for metallographic testing and hardness testing. The flow chart of the process is shown in Figure 3.

Sample preparation included metallographic preparation, cutting in the axial direction to include both base materials and the joint, technological preparation by grinding with sandpaper, surface polishing with diamond-based pastes, as well as final polishing with an appropriate solution.

Sample preparation also included cutting samples in the joint zone. The exception is that the crown of extruded material (mushroom) was first removed by cutting and then pieces were cut in the axial direction that included both materials and the joint. Those samples were further technologically prepared by grinding with sandpaper of fineness R340–R3000, and then the surfaces were polished with diamond-based pastes of a certain granulation (for example 6, 3, 2 μm). For final polishing, an appropriate solution is used.

**Figure 4:**
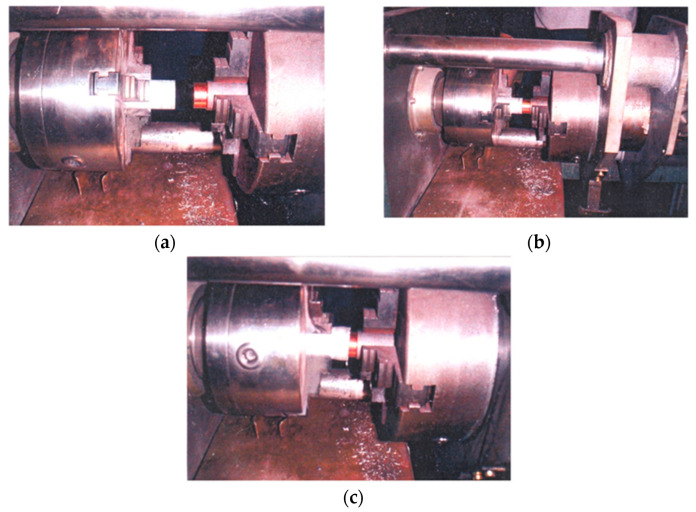
The actual appearance of the experimental procedure. (**a**) the first phase—contact between Al and Cu surfaces established; (**b**) the second phase—intense friction; (**c**) the third phase—the compression phase—joint formation.

The experimental process is carried out on the prepared samples in three phases (as shown in Figure 4). The first phase (a) involves placing the parts in the jaws of the machine and establishing contact between the front surfaces of the aluminum and copper samples; in the second phase (b) the aluminum part rotates, and the copper part performs the axial movement so that intense friction occurs. The third phase (c) is the compression phase where an axial force is introduced, intense plastic deformation occurs and a crown or the so-called “mushroom” is formed. Thus, the joint is formed.

**Figure 5:**
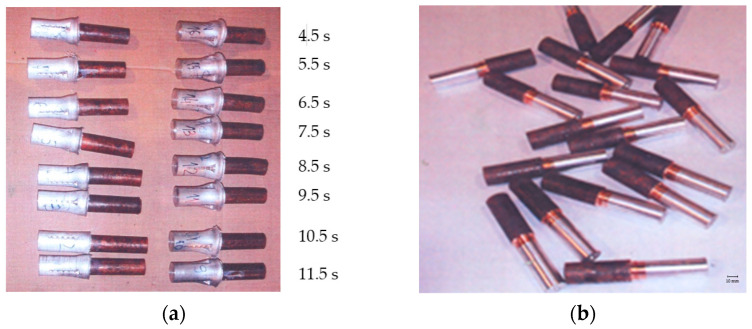
(**a**) Appearance of the friction-welded samples as a function of the friction time; (**b**) friction-welded joints after the machining.

The friction time (t) is the time required for the contact surfaces to heat up to reach the maximum temperature necessary for the welding process to begin. As an important parameter in the friction welding process, the friction time depends on other factors such as the properties of the base materials (strength, thermal conductivity, ductility, etc.), friction speed, friction pressure, shape and dimensions of the welded parts, etc. [14]. When different metals are being welded, the friction time, along with other welding parameters, is often chosen based on the material of a lower strength. The friction time is measured from the beginning of the second phase until the moment when the pneumatic brake instantly stops the rotation of the aluminum part. The finished welded joints are shown in Figure 5a.

In the next step of the process, the extruded material was completely removed by machining. Then, the joint line became visible on the machined samples, which otherwise cannot be observed under the “mushroom”, as the extruded material covers not only the joint line but the entire joining zone, as well. Figure 5b shows the samples after machining by cutting.

In the experimental section of this research, changes in the dimensions of welded samples were monitored both on the aluminum and copper parts, as well as the overall change. The main parameters to be considered in friction welding are technological parameters: speed n, friction pressure p_t_, compression pressure p_c_, friction time, as well as geometric parameters, i.e., the initial dimensions of the samples. The concrete values of those parameters in this experiment were: n = 2000 rpm, p_t_ = 84 MPa, p_c_ = 200 MPa, t = 4.5–11.5 s. The cylindrical samples’ initial dimensions were a diameter of 30 mm and a length of 90 mm for the aluminum part, and a diameter of 30 mm and a length of 100 mm for the copper part.

The newly formed dimensions were measured on these processed samples, including the overall length of the sample (L_1t_), the lengths of each of the Al and Cu elements after welding (L_1_), and the crown-shaped diameter (d_v_). Then, the shortenings of each of the Al and Cu elements (ΔL_1_), as well as the total shortening of the welded sample (ΔL_1t_), were determined, all as a function of the friction time. The results are presented in Table 2.

**Figure 6:**
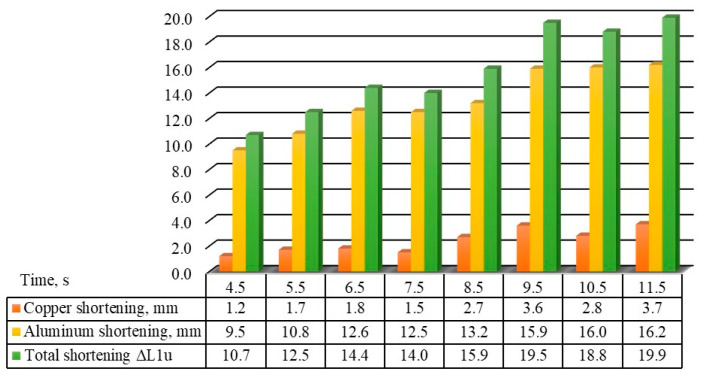
The shortening of samples as a function of the friction time.

Thermomechanical effects in the weld zone led to intense and rapid plastic deformation, resulting in an axial shortening of the welded elements. The magnitude of axial shortening depends on several factors related to the welded material and its geometric characteristics, as well as the welding process parameters (pressure, welding speed, welding time, etc.) [17]. The value and rate of axial deformation increase with increasing friction time and pressure, and for each material, their optimal values were experimentally determined. In comparison to the optimal values, small values of axial shortening lead to incomplete and poor-quality joints, as there may be the occurrence of the so-called circular unwelded areas. Larger values of axial shortening result in increased material consumption during the welding. Based on the expected axial deformation (shortening), the final lengths of the welded parts were determined. The shortening as a function of the friction time is presented in the histogram in Figure 6.

**Figure 7:**
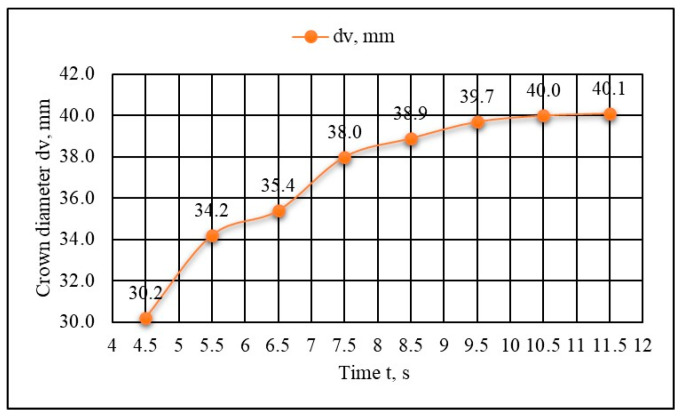
Crown diameter (d_v_) dependence on the welding time.

There is a similar relationship between the friction time and lateral deformation, which is more difficult to monitor due to the accumulation of aluminum and the formation of the “mushroom”. The diameter of the “mushroom” depends on the friction time as well. As the friction time increases, the diameter of the extruded material (aluminum) or “mushroom” also increases (Figure 7).

Depending on the friction time, as well as the friction pressure and tension, the crown diameter increases from 2 to 40%, compared to the initial diameter of the aluminum element.

### 3.5. Microstructure Examinations of the Welded Joints

**Figure 8:**
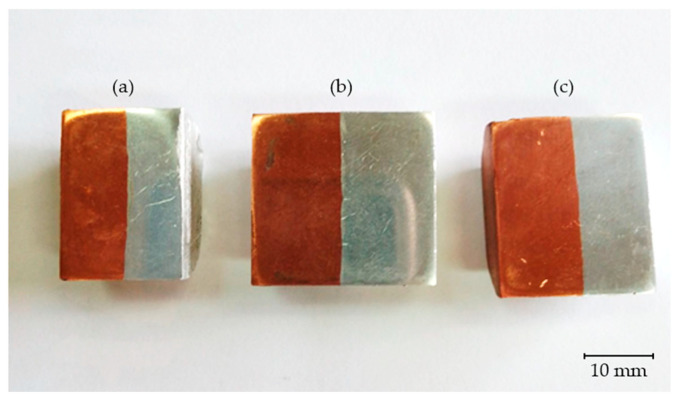
Al-Cu joint samples for microstructural investigations. Friction times 6.5 s (**a**), 7.5 s (**b**), 9.5 s (**c**).

Figure 8 shows the samples prepared for microscopic examinations and hardness measurements. The samples differ based on the friction time.

**Figure 9:**
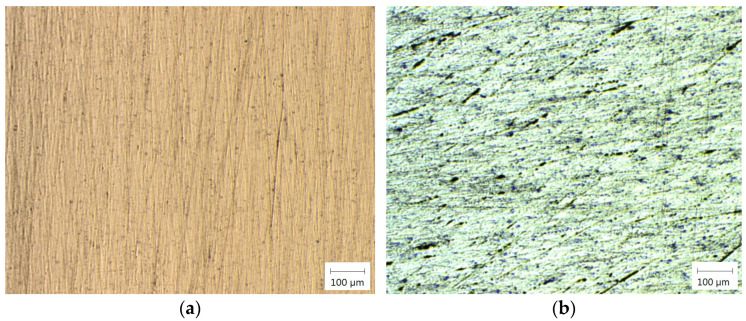
Microstructure of (**a**) copper; (**b**) aluminum. The microstructure of the joint zone is presented in Figure 8, for different welding times (6.5, 7.5, 9.5 s).

Figure 9 shows the microstructure of the base material of copper and aluminum.

**Figure 10:**
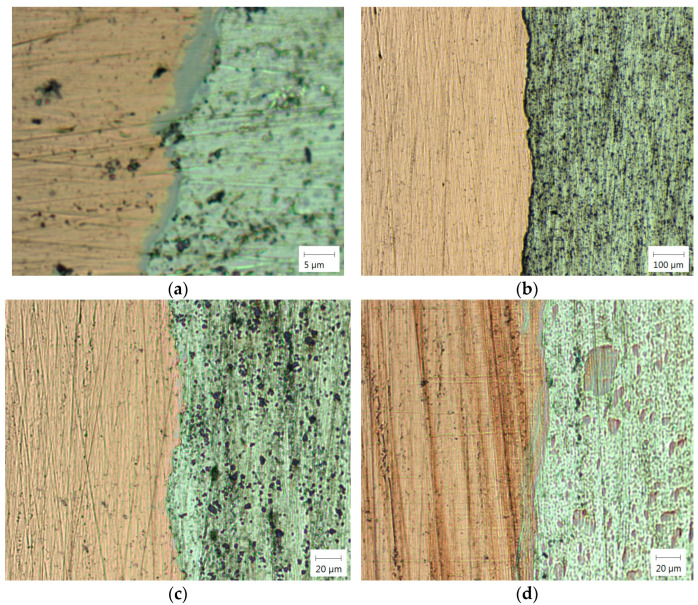
Microstructure of the Al-Cu joint zone: (**a**) friction time 7.5 s; (**b**,**c**) friction time 9.5 s; (**d**) friction time 6.5 s.

In Figure 10a, an immediate mixing zone is observed along the joint interface, where the diffusion is manifested as interdiffusion of the two base metals, i.e., the diffusion of copper into aluminum and vice versa. Immediately next to the intermetallic layer, a thermo-mechanically affected zone on the Al side is identified, which is located near the weld center. The grains are equiaxed with the assumption that they are recrystallized due to accumulated strain energy.

Since the deformation in the heat-affected zone influences changes in microstructure, it is essential to determine the hardness and check for the potential presence of the brittle phases (θ—CuAl_2_, and δ—Cu_9_Al_4_). This experiment included hardness measurements on the welded samples, with measurements taken at specific points. The results of the hardness distribution are presented in Table 3.

As a result of thermo-mechanical effects during the welding process in the joint zone and the heat-affected zone (HAZ), as anticipated, there has been a change in hardness. The hardness was measured by the Vickers method. The measured hardness values indicate a significant increase in the joint zone on the aluminum side. This increase is attributed to the deformation process and the presence of intermetallic phases. In contrast, the copper part shows a certain stagnation and a decrease in hardness values near and within the joint zone, due to the formation of a softening zone, Table 3. This heterogeneity in hardness distribution becomes more pronounced as the friction time and welding speed change. Moreover, Zhu et al. [13] studied the effect of rotational speed on continuous drive friction-welded joints between 6061-T6 aluminum and copper. They found that higher rotational speeds refined grains in the dynamically recrystallized zone (DRZ) and enhanced element inter-diffusion at the bonding surface due to heat and force. The Crystal’s microhardness at the bonding surface increased due to intermetallic compounds (IMCs), while softening zones formed on both sides. Besides that, due to the thermal influence, the thermo-mechanical affected zone undergoes dissolution of precipitates and has a grain size coarser than the base material due to the thermo-mechanic effect of the welding process. If the grain size is coarser, the total area of grain boundary per unit volume (that opposes electron mobility) is smaller, so, the electron mobility is easier to achieve and consequently, the electrical conductivity increases to about 17% relative to the base material [28].

## 4. Numerical Simulation

Numerical simulation of the friction welding process was performed using the *Simufact forming 15.0* software. The software employs the finite volume method and the finite element method, specifically the Dytran and Marc solvers. The governing equations of the FRW process are well known and it was not necessary to incorporate them in the text (Coulomb friction law, the von Mises yield condition, associated flow rule using the von Mises yield function, isotropic, bilinear hardening rule, etc.) [30,31,32,33]. The *Simufact forming* software has a special module *Pressure welding* in which the process type *Friction welding* was selected and the finite element method to simulate the friction welding process in this study. Since the parts are axisymmetric, a 2D simulation was performed. In this way, the calculation time was drastically reduced compared to the 3D simulation. The input parameters for FE simulation, presented in Table 3, provide convergence solutions and quality results. Since in the numerical simulation within this software, only one workpiece can be used, the other part (the aluminum one) was defined as a deformable tool. In the 2D simulation, two types of meshers for Al and Cu specimens were used: Advancing front quad and Quadtree. Quadtree has slightly better capabilities for creating a finer mesh. Selected element size was 0.3 mm as in the deformation zones, where larger strains occur, the mesh was refined using the refinement box with refinement level 1. Refinement boxes were defined at the contact points on both samples. The contact table between the elements was defined according to the software recommendations [34]. Both meshers use the Quads (10) finite element of square shape, which is intended for numerical simulations when the workpiece is axisymmetric.

Three values of the friction time were applied as in laboratory experiments (4.5 s, 7.5 s, and 11.5 s), representing the minimum, average, and maximum contact durations at the beginning of the process.

Since in the numerical simulation within this software, only one workpiece can be used, the other part (the aluminum one) was defined as a deformable tool. The simulation parameters are presented in Table 4. Refinement boxes were defined at the contact points on both samples. 

The comparison of results obtained experimentally and numerically is presented in Table 5. The deviations are acceptable, except in the case of 11.5 s, where the dimensions of the part differ by nearly 2 mm.

**Figure 11:**
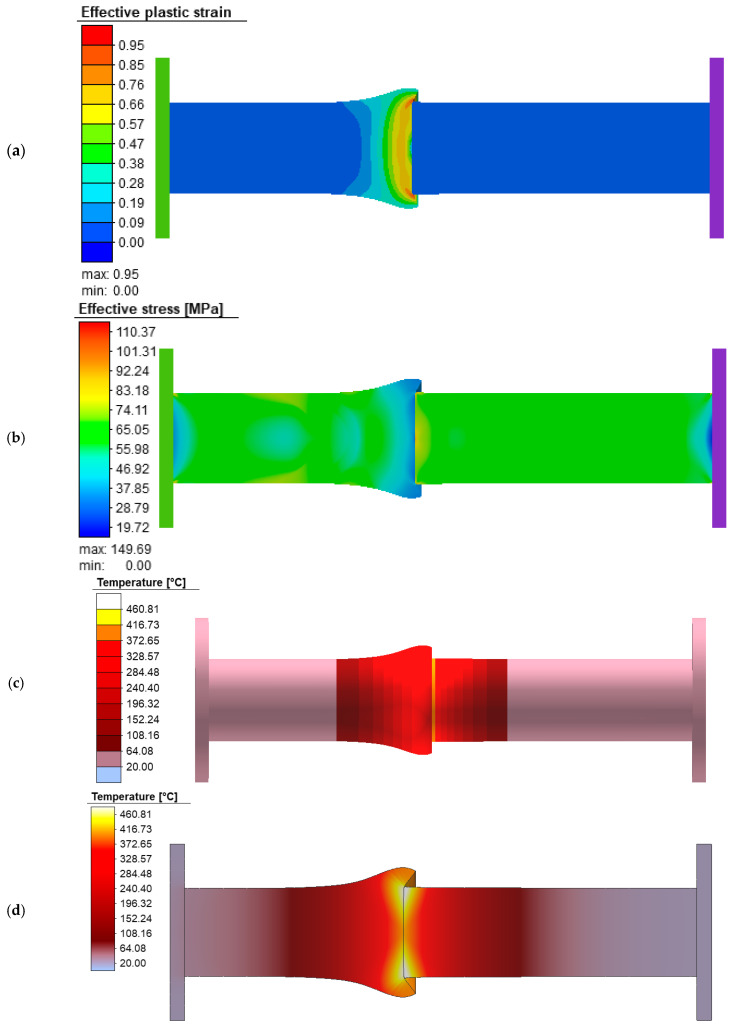
Numerical simulation results (friction time 4.5 s): (**a**) the cross-section effective plastic strain distribution; (**b**) the cross-section effective stress distribution; (**c**) workpiece temperature; (**d**) the cross-section temperature distribution.

The results of the numerical simulation are presented in Figure 11. The deviations observed between the experimental and numerical results can be explained by the quality of the input data. In the numerical simulation, materials from the software’s database were used, where each material is defined by a flow curve in analytical form, along with other characteristics, such as the heat transfer coefficients, etc. Given that the properties of the used materials were not checked by the tensile tests, the data from the software’s database were the only available ones. It was realistic to expect that there should be differences in characteristics between the materials used in the experiment and those defined in the software’s database.

The numerical results are shown in Figure 11. Figure 9a shows the results of the effective plastic strain, while Figure 11b shows the values of the effective stress and its value goes up to 150 MPa. Figure 11c shows the temperature of the workpiece, which in the case of a friction time of 4.5 s is about 460 °C and in Figure 11d the result of the temperature in the cross-section is presented.

The results of the numerical simulation show that after 4 s the highest value of the effective stress is 70 MPa and the temperature near the contact of the two parts is 257 °C. After the start of the deformation of the aluminum part, the temperature and effective stress value continue to increase. The highest value of the effective stress is on the copper sample, it occurs for the working stroke value of 9.24 mm and it amounts to 149 MPa.

## 5. Discussion of Results and Conclusions

The results of this study shed light on the nature of the Al-Cu joint obtained through friction welding. With the increase in friction time, axial deformation (shortening) of both the aluminum and copper elements increases. Due to the properties of aluminum, that part deforms more than the copper one, i.e., the shortening of the aluminum part was 4.2 to 10.3 times greater than that of the copper part, within the friction time range of 4.5 s to 11.5 s. For the shortest friction time of 4.5 s, the shortening of the copper part was 1.2 mm and of the aluminum part was 9.5 mm, while for the longest friction time, the copper part shortening was 3.7 mm and the aluminum part shortening was 16.2 mm. When the friction time increases 3 times, the shortening of the copper part increases 3 times, while this ratio does not apply to the aluminum part. However, overall shortening increases 1.8 times for the friction time interval (4.5 s to 11.5 s). These facts are significant due to the initial dimensions of the components of both materials and their preparation.

From a comprehensive analysis of the experiment, it was determined that, depending on the relevant parameters of the friction welding process, the optimal welding time in this case is 6.5–11.5 s.

On the other hand, the axial deformation, both of individual parts and in total, affects the mechanical and microstructural properties of the joint. The lateral deformation gradually increases with friction time increase; for a friction time of 4.5 s, the diameter of the crown (d_v_) is 30.2 mm, and for a friction time of 11.5 s, the diameter of the crown has increased to 40.1 mm, indicating a permanent deformation of about 34%.

Friction stir welding (FSW) is an efficient method for joining components made of Al and Cu. The combination of friction stir welding of Cu and Al has been relatively well researched, with numerous experiments conducted to achieve optimal results for all parameters. The application of these parameters can produce high-quality joints. Different metals reach plastic deformation due to the applied friction, creating a narrow heat-affected zone, thus preserving the molecular structure of the components.

Plastic deformation affects the mechanical and microstructural properties of the welded joint in such a way that a lower degree of plastic deformation causes (and this is again related to the developed heat released by the friction of the sample surfaces) limited diffusion of atoms in the contact layer, because it is necessary to increase the contact surface in microvolumes of the material. Sufficient mixing of the materials is not achieved, and therefore good metallurgical bonding is not created and defects may occur at the interface. With an increase in the degree of plastic deformation, the deformation of aluminum increases. The reaction and diffusion of aluminum and copper are more adequate, which forms a good metallurgical bond in the welded joint, due to which the tensile strength of the joint is improved. Newly formed phases and microstructural components, as a result of thermodynamic effects, also depend on axial deformation, especially their concentration, quantity and arrangement in the joint.

Intermetallic phases and microstructural defects are accompanying phenomena in the joint zone as a normal consequence of the heat input and the effect of plastic deformations. Phases such as Al_2_Cu, Al_4_Cu_9_ are formed at lower values of the friction time, while the AlCu phase is formed at higher values of the welding time. The thickness of the intermetallic layer increases exponentially with increasing welding time. Process conditions, such as compression pressure, welding time and the temperature developed in the interlayer, contribute in a corresponding way to the formation of the interdiffusion layer, as well as the appearance and development of the mentioned phases. Since Al has a lower melting point and is a more ductile material than Cu, that leads to the fact that Al reaches a state of plastic deformation earlier and begins to separate and mix first, which leads to greater thicknesses of the Al_2_Cu phase layer, not only in the narrow joint zone but in the heat affected zone (HAZ), as well. Aluminum has also a lower strength than copper, so it experiences a greater degree of plastic deformation, which is manifested by the material being ejected around the rim (“mushroom”).

The welding time affects the growth of the interdiffusion layer. With the growth of the interdiffusion layer, there is a possibility of the formation of inhomogeneities in the weld, and therefore it is necessary to prescribe the welding time strictly according to the set parameters, to avoid the production of a joint of a strength that is smaller than the required value.

The friction time has a certain effect on the hardness of the welded joint, with hardness increasing slightly with increasing friction time. To determine the precise nature of this dependence would require more extensive research.

It has been proven that a short friction time does not provide sufficient heat input, which means that the plastic flow of the metal layers does not start, and the joint does not even form, or has a very low strength. On the other hand, excessive welding time leads to a large heat input, which creates conditions for the formation of the intermetallic phases in the welding zone. The welding time also affects the amount of intermetallic phases, which significantly affect the mechanical properties of the welded joint and can cause brittleness in the weld area.

The small deviation between the experimental and numerical results confirms that the numerical model is reliable and that it is possible to analyze the friction welding process using numerical simulations. The small deviations observed between the experimental and numerical results can be explained by the quality of the input data related to material properties. In the numerical simulation, the materials’ library in *Simufact forming* software was used, where the referent materials in this study are defined by a flow curve in analytical form, along with other characteristics, such as the heat transfer coefficients, etc. Given that the properties of the used materials were not determined by the tensile tests, the data from the materials’ library was the only available one. It was realistic to expect that there should be differences in characteristics between the materials used in the experiment and those defined in the software’s database.

During the experiment, there was no technical possibility to record the temperature field, so it is not possible to make a comparison of temperature fields with the results obtained by numerical simulation. However, based on the good agreement of other, primarily geometric results, the results for the temperature field obtained numerically can be considered relevant.

To achieve a higher-quality weld, it is necessary, among other relevant parameters such as friction pressure, forging pressure, number of rotations, etc., to define the welding time as accurately as possible. The ideal welding time is difficult to determine because it depends on the welded metals. In the case of welding the two different metals, the welding time varies for each metal. In addition, since the welding time is divided into the time of action of the working pressure and the time of action of the compressing pressure, the optimal values of the time are different for each metal, as they also depend on other processes and other parameters. Therefore, it is best to choose the optimal values empirically. A short welding time leads to insufficient heat release, resulting in the reduced plastic flow of the metal, so the joint may not even form or may be of insufficient strength. Excessive welding time leads to excessive heat release, resulting in increased formation of intermetallic phases in the weld zone and HAZ, which, besides causing brittleness of the joint, can change its electrical and thermal conductivity, as well.

Considering that such structural elements prioritize electrical conductivity for their intended purpose, mechanical properties are of lesser importance, as they do not carry loads during operation. Electrical conductivity values depend, among other factors, on the size of metal grains and the concentration of certain defects in the crystal lattice. Decreasing the grain size results in reduced electrical conductivity due to an increase in the number of grain boundaries, which act as barriers to electron movement. Increasing the grain size in the HAZ can lead to enhanced electrical conductivity.

Based on the foregoing, it can be concluded that the friction time between these two metals significantly affects the properties and quality of the welded joint. The friction time, compression time, and all the other relevant parameters are best selected within optimal ranges, depending on all the specified geometrical, operational, and other conditions, to achieve high-quality joints that fully meet their intended function.

The application of scientific results in industrial practice is significant because it contributes to the advancement and improvement of industrial processes. For example, achieving higher quality compounds, as well as a quantitative contribution in terms of greater production per unit of time. In addition, the application of optimal solutions provides not only technological benefits but also economic effects.

Future work would be based on research into the application of other existing numerical models to examples of friction welding, not only conventional welding but FSW welding, as well. Research would also be focused on finding a mathematical model that would define the joining process itself. For example, the equation of the flow of material in the appropriate direction from the beginning of friction until the permanent deformation of each material is achieved. Additionally, the SEM analysis of the microstructure in the joint zone would be carried out, as well as research into some other mechanical properties.

## Figures and Tables

**Table 1:** Properties of the materials used.

Property	Al	Cu
Melting point, °C	660.4	1083
Heat conductivity, W/mK	222	395
Density, g/cm^3^	2.7	8.96
Coefficient of linear expansion, 1/°C	23.9	16.5
Tensile strength, MPa	50–80	150
Hardness, HB	15–20	25
Modulus of elasticity, MPa	71,000	127,000
Elongation, %	35–45	52
Plasticity	Very well	Very well

**Table 2:** Measurement results of the dimensional changes in samples after the friction welding.

Friction Time	Dimensions After the Friction Welding						
t, s	L_1_, mm		ΔL_1_, mm		ΔL_1t_, mm	ΔL_1t_, %	d_v_, mm
	Al	Cu	Al	Cu			
4.5	80.5	98.8	9.5	1.2	10.7	5.6	30.2
5.5	79.2	98.3	10.8	1.7	12.5	6.5	34.2
6.5	77.4	98.2	12.6	1.8	14.4	7.6	35.4
7.5	77.5	98.5	12.5	1.5	14	7.4	38
8.5	76.8	97.3	13.2	2.7	15.9	8.3	38.9
9.5	74.1	96.4	15.9	3.6	19.5	10.2	39.7
10.5	74	97.2	16	2.8	18.8	9.8	40
11.5	73.8	96.3	16.2	3.7	19.9	10.4	40.1

**Table 3:** Hardness distribution along the axis of the sample for a friction time of 9.5 s.

	Cu					Al					
Hardness, HV10	83.8	84	71	62.7	64	48.2	43.2	40	31.2	30.3	30
Distance from the joint line, mm	−5	−4	−3	−2	−1	0	1	2	3	4	5

**Table 4:** Numerical simulation parameters.

Parameter	Value
Simulation type	2D
Mesher	Aluminum specimen—Advancing front quad Copper specimen—Quadtree
Finite element type and dimension	Aluminum specimen: Quads (10)—0.3 mm Element count: 16,548 Copper specimen: Quads (10)—0.3 mm Element count: 23,200
Friction	Coulomb µ = 0.1

**Table 5:** Comparison of experimental and numerical results on welded parts dimensions, mm.

Friction Time, s	Experimental Results		Numerical Results		Temperature (Numerical Simulation), °C
	Al	Cu	Al	Cu	
4.5	80.50	98.80	80.54	98.77	460
7.5	77.50	98.50	77.71	98.36	484
11.5	73.80	96.30	73.59	98.30	522

## Data Availability

The original contributions presented in this study are included in the article. Further inquiries can be directed to the corresponding authors.
